# How Long Would You Like to Live? A 25-year Prospective Observation of the Association Between Desired Longevity and Mortality

**DOI:** 10.2188/jea.JE20210493

**Published:** 2023-09-05

**Authors:** Yuta Yokokawa, Toshimasa Sone, Sanae Matsuyama, Yukai Lu, Yumi Sugawara, Akira Fukao, Ichiro Tsuji

**Affiliations:** 1 Division of Epidemiology, Department of Health Informatics and Public Health, Tohoku University School of Public Health, Graduate School of Medicine, Sendai, Japan; 2 Miyagi Cancer Society, Sendai, Japan

**Keywords:** desired longevity, lifestyle behaviors, mediation analysis

## Abstract

**Background:**

Desired longevity represents how strongly people esteem possible extensions of their own lifetime. The association between desired longevity and mortality risk has been reported in only one prospective study, which examined a small sample of older participants. We aimed to examine the hypothesis that desired longevity at middle-age predicted long-term survival.

**Methods:**

In the prospective cohort study, residents aged 40–64 years were asked how long they would like to live and asked to choose one from three options: longer than, as long as, or shorter than the life expectancy. We used Cox proportional hazards model to calculate the hazard ratios (HRs) and 95% confidence intervals (CIs) for all-cause and cause-specific mortality according to the three groups for desired longevity, treating the “longer than” group as the reference. We conducted mediation analysis to investigate the mechanism for the association between desired longevity and mortality.

**Results:**

We recruited 39,902 residents to the study. Risk of all-cause mortality was significantly higher in the “shorter than” group (HR 1.12; 95% CI, 1.04–1.21). The association was independent of sex, age, marital status, education, medical history, and health status. Regarding cause of death, mortality risk of cancer (HR 1.14; 95% CI, 1.00–1.29) and suicide (HR 2.15; 95% CI, 1.37–3.38) were also higher in the “shorter than” group. The unhealthy lifestyle mediated this association with all-cause mortality by 30.4%.

**Conclusion:**

Shorter desired longevity was significantly associated with an increased risk of all-cause mortality, and mortality from cancer and suicide. Lifestyle behaviors particularly mediated this association.

## INTRODUCTION

The recent extension of life expectancy accompanied not only an increase in the years of healthy living, but also in the years spent with disease or disability.^1^^,^^2^ Longevity itself is no longer the ideal goal for everyone, and preference for longevity has become highly individual.^1^^,^^3^^–^^5^ With this changing trend, a new research field in desired longevity has been emerging.^3^^–^^7^

Desired longevity is usually determined by asking “how long would you like to live?” or “to what age would you like to live?”. Desired longevity is reported to represent how strongly people esteem possible extensions of their own lifetime.^4^^,^^8^ Several studies have measured desired longevity among varying settings and populations, and examined the determinants and consequences of desired longevity.^3^^–^^6^ Desired longevity has been associated with positive psychological wellbeing, such as happiness, optimism, life satisfaction, purpose in life, and so forth.^9^^,^^10^ Although the association between positive psychological wellbeing and lower risks of mortality and morbidity has been verified by prospective cohort studies,^11^^–^^16^ evidence on the association between desired longevity and mortality is limited.^17^ There is only one prospective study, which reported that shorter desired longevity was significantly associated with an increased risk of all-cause mortality. This study was, however, limited because it examined only a small sample (*n* = 283) of older (aged 75–90 years) participants, followed for 10 years, and only adjusted for age, sex, comorbidity, and depressive feelings. Thus, reverse causality and the effects of unmeasured confounders could not be fully ruled out.

The purpose of this study was to examine the hypothesis that desired longevity at middle-age predicted long-term survival independent of possible confounders or comorbidity. For this purpose, we prospectively followed about 40,000 participants aged 40 to 64 years in Japan for about 25 years. We also conducted mediation analysis in order to examine the role of health-related lifestyle in the association between desired longevity and mortality.

## METHODS

### Study design and participants

This study was based on the Miyagi Cohort Study, details of which have been reported previously.^18^^,^^19^ In brief, from June through August 1990, all 51,921 residents (25,279 men and 26,642 women) aged 40–64 years living in 14 municipalities, which were randomly selected from 62 municipalities in Miyagi Prefecture, Northeastern Japan, were entered into a cohort. A self-administered questionnaire asking about sociodemographic factors, lifestyle, and health was delivered to and collected from individuals’ residences by each municipal government. The response rate for the questionnaire was 91.7% (*n* = 47,604). The study protocol was approved by the Ethical Committee of Tohoku University Graduate School of Medicine (approval number: 2014-1-838). We considered that the return of self-administered questionnaires signed by the participants implied their consent to participate in the study.

### Measurements

We focused on the association between desired longevity and mortality risk. Desired longevity was defined as length of life which participants wanted to live. Participants were asked in the questionnaire: “How long would you like to live?”; possible responses were “I would like to live as long as possible”, “living for life expectancy (in 1990, 76 years for men, 81 years for women^20^) is good enough”, and “I may live shorter than life expectancy”. According to the answer, we classified participants into three desired longevity groups, “longer than”, “average”, and “shorter than” the life expectancy of Japan in 1990.

As in a previous study,^21^ we regarded the following variables as potential confounders (base model): age (continuous variable), sex (men or women), marital status (married, divorced/widowed, or single) and education (in school until age ≤15 years, age 16–18 years, or age ≥19 years).

We considered the following variables as possible mediators for the association between desired longevity and mortality risk: body mass index (BMI) in kilograms per meter squared (<18.5, 18.5–24.9, or ≥25.0 kg/m^2^), smoking status (never smokers or ever smokers), drinking status (never drinkers or ever drinkers), sleep duration (≤6 hours, 7–8 hours, or ≥9 hours), time spent walking per day (<1 hour or ≥1 hour), and eating breakfast (yes or no). These variables were chosen because these lifestyle behaviors were shown to be associated with mortality in the Alameda County Study.^22^^,^^23^

### Outcomes

Primary outcomes were mortality from all causes and the following cause-specific mortality: ischemic heart disease (IHD), stroke, cancer, pneumonia, accident, and suicide. The cause of death was classified according to the International Classification of Diseases (ICD); we used the 9th revision between June 1, 1990, and December 31, 1998^25^ and the 10th revision between January 1, 1999, and March 31, 2015.^26^ Deaths from each cause was identified as follows: IHD (ICD-9: 410–414 or ICD-10: I20–I25), stroke (ICD-9: 430–438 or ICD-10: I60–I69), cancer (ICD-9: 140–208, 230–234 or ICD-10: C, D00–D09), pneumonia (ICD-9: 480–489 or ICD-10: J10–18), accident (ICD-9: 1800–1949, 1960–1999 or ICD-10: V, W, Y, X00–59, X85–99), and suicide (ICD-9: 1950–1959 or ICD-10: X60–84). We defined cardiovascular disease as a combination of IHD and stroke.

### Follow-up

To follow-up the participants for mortality and migration, a Follow-up Committee was established,^24^ which consisted of the Miyagi Cancer Society, the Community Health Divisions of all 14 municipalities, the Department of Health and Welfare of Miyagi Prefectural Government, and the Division of Epidemiology, Tohoku University Graduate School of Medicine. The Committee periodically reviewed the Residential Registration Record of each municipality and the death certificates with permission from the Ministry of Health, Labour and Welfare, Japan. Through this review, we identified participants who had either died or emigrated from June 1, 1990 to March 31, 2015, and we reviewed the causes of death for the decedents. We discontinued the follow-up of those who had moved away from the study municipalities, because the Committee was unable to review the Residential Registration Record outside the study area. We counted person-years of follow-up for each participant from June 1, 1990 until the date of death, emigration, or March 31, 2015, whichever occurred first.

Of the 47,604 participants, we excluded 2,137 participants who had a history of cancer, stroke, or myocardial infarction at baseline to limit reverse causality. We also excluded 5,565 participants who had not answered the question about desired longevity. Finally, 39,902 participants (19,443 men and 20,459 women) were used for this analysis (Figure 01). During almost 25 years from June 1, 1990 to March 31, 2015, 3,606 participants (9.0%) were lost to follow-up due to migration and thus, the follow-up rate was 91.0%. During the 870,688 person-years of follow-up, 8,998 participants (22.6%) died.

**Figure 1. fig01:**
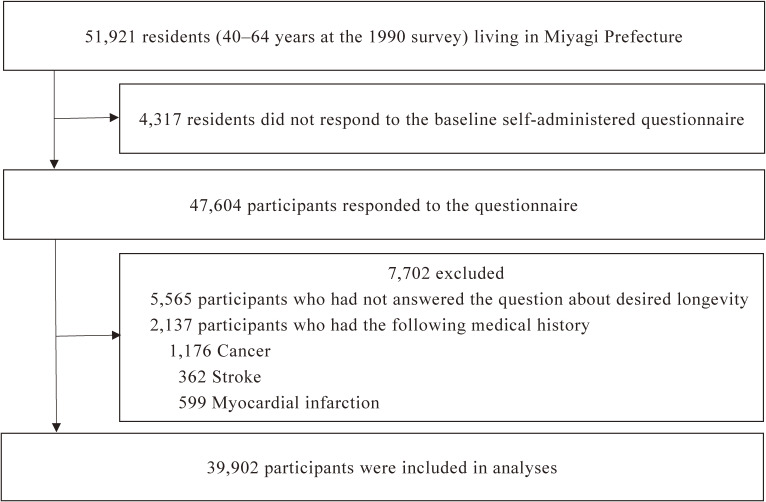
Participants flow chart

### Statistical analysis

We assessed the differences in baseline characteristics between three desired longevity groups, using analysis of variance for continuous variables and chi-square tests for categorical variables. To estimate cumulative mortality, we used the Kaplan-Meier method. We used Cox proportional hazards model to calculate the hazard ratios (HRs) and 95% confidence intervals (CIs) for mortality from all causes and cause-specific mortality according to the three groups of desired longevity, treating the “longer than” group as the reference, after adjustment for potential confounders. The proportionality assumption was examined using the Schoenfeld residuals test.

In addition, to assess whether the association between desired longevity and mortality differed by potential confounders, we stratified the participants according to the potential confounders: age (40–49 years, 50–59 years, or ≥60 years), sex (men or women), marital status (married or unmarried) and education (in school until age ≤15 years or age ≥16 years). We also stratified the participants according to past medical history of hypertension and diabetes.

Furthermore, we repeated the analyses after including 2,137 participants who had a history of cancer, stroke, or myocardial infarction at baseline. We also repeated the analyses after excluding all deaths that occurred within the first 2 years of follow-up because participants who died during this period might have been in poor health at baseline.

We performed mediation analysis in order to investigate the mechanism of the association between desired longevity and mortality. We evaluated the proportion of mediating effects of lifestyle behaviors on the association between desired longevity and mortality. In mediation analysis, we first calculated HRs and 95% CIs of the base model. Second, we added each variable of lifestyle behaviors (BMI, smoking status, drinking status, sleep duration, time spent walking, and eating breakfast) to the base model, and calculated the proportion of mediating effect and its 95% CI using the publicity available %Mediate macro.^27^ To test the effect of each potential mediator, we used the Baron and Kenny causal steps approach.^28^ We also estimated logistic regression to calculate path coefficients (total effect, direct effect, indirect effect) and performed the Sobel test.^29^ We repeated mediation analyses after stratifying age and sex. Statistical analyses were performed using the SAS software package (version 9.4: SAS Institute Inc., Cary, NC, USA). All reported *P* values were two-sided and were considered statistically significant if <0.05.

## RESULTS

Regarding the question “How long would you like to live?”, 13,198 participants (33.1%) answered “I would like to live as long as possible” (the “longer than” group), 21,829 participants (54.7%) answered “living for life expectancy is good enough” (the “average” group), and 4,875 participants (12.2%) answered “I may live shorter than life expectancy” (the “shorter than” group). Baseline characteristics according to desired longevity categories are shown in Table 1. Compared with the “longer than” group, the “shorter than” group was younger, less likely to be men, and had a longer educational duration. Regarding lifestyle, stratified by gender, the “shorter than” group was more likely to smoke, drink and less likely to sleep, walk, and eat breakfast (eTable 1).

**Table 1.** Demographic characteristics of sample according to the desired longevity (*n* = 39,902)

	Desired longevity			*P*-value

	Longer than	Average	Shorter than	
Number of participants	13,198	21,829	4,875	
Age, years, mean (SD)	52.7 (7.6)	51.5 (7.5)	49.8 (7.1)	<0.001
Men, %	56.3	47.4	34.2	<0.001
Marital status, %				<0.001
Married	90.0	90.9	87.2	
Divorced/widowed	6.6	6.6	8.4	
Single	3.4	2.5	4.4	
Education, %				<0.001
≤15 years	43.4	37.9	34.5	
16–18 years	44.5	47.6	50.3	
≥19 years	12.1	14.5	15.2	

SD, standard deviation.

The association between desired longevity and all-cause mortality is shown in Table 2. When we adjusted for age and sex, the “shorter than” group was associated with a 12% higher hazard of dying over the follow-up period than the “longer than” group (HR 1.12; 95% CI, 1.04–1.21). This 12% higher hazard was also present when we adjusted for age, sex, marital status and education (HR 1.12; 95% CI, 1.04–1.21).

**Table 2.** Hazard ratios and 95% confidence intervals for all-cause mortality according to desired longevity (*n* = 39,902)

	Desired longevity		

	Longer than	Average	Shorter than
Number of participants	13,198	21,829	4,875
Person-year of follow-up	286,841	478,457	105,390
Number of deaths	3,396	4,717	885
Age and sex adjusted HR (95% CI)	1.00 (ref.)	0.99 (0.95–1.04)	1.12 (1.04–1.21)
Base model HR (95% CI)	1.00 (ref.)	1.00 (0.96–1.05)	1.12 (1.04–1.21)

CI, confidence interval; HR, hazard ratio.

Base model hazard ratio: adjusted for age (continuous variable), sex (men or women), marital status (married, divorced/widowed, or single), education (in school until age ≤15 years, age 16–18 years, or age ≥19 years).

The associations between desired longevity and all-cause mortality were observed in every subgroup according to age, sex, marital status, and education (Table 3). The results revealed that all-cause mortality risk was consistently higher for the “shorter than” group whether they were younger or older, men or women, married or not, and highly educated or not, although it was not significant.

**Table 3.** Base model hazard ratios and 95% confidence intervals for all-cause mortality according to desired longevity stratified by age, sex, marital status, and education

	Desired longevity			*P* for interaction

	Longer than	Average	Shorter than	
Age				
40–49 years	1.00 (ref.)	0.96 (0.86–1.07)	1.11 (0.96–1.29)	0.25
50–59 years	1.00 (ref.)	0.98 (0.92–1.06)	1.11 (0.99–1.24)	
≥60 years	1.00 (ref.)	1.05 (0.98–1.12)	1.13 (0.99–1.29)	
Sex				
Men	1.00 (ref.)	1.04 (0.98–1.10)	1.19 (1.07–1.32)	0.049
Women	1.00 (ref.)	0.94 (0.87–1.01)	1.04 (0.93–1.16)	
Marital status				
Married	1.00 (ref.)	0.99 (0.94–1.04)	1.11 (1.02–1.21)	0.19
Others^a^	1.00 (ref.)	1.12 (0.97–1.28)	1.27 (1.04–1.56)	
Education				
≤15 years	1.00 (ref.)	1.02 (0.96–1.09)	1.09 (0.97–1.23)	0.96
≥16 years	1.00 (ref.)	0.99 (0.93–1.06)	1.15 (1.04–1.28)	

^a^Others: divorced, widowed, or single.

Base model hazard ratio: adjusted for the following variables (except for stratifying variables); age (continuous variable), sex (men or women), marital status (married, divorced/widowed, or single), education (in school until age ≤15 years, age 16–18 years, or age ≥19 years).

The association between desired longevity and cause-specific mortality is shown in Table 4. Compared with the “longer than” group, the “shorter than” group was associated with a 14% higher hazard of dying of cancer (HR 1.14; 95% CI, 1.00–1.29) and 115% higher hazard of dying of suicide (HR 2.15; 95% CI, 1.37–3.38) when we adjusted for age, sex, marital status, and education. Regarding other causes of mortality, the “shorter than” group was similarly associated with a higher hazard of dying, but this was not significant.

**Table 4.** Base model hazard ratios and 95% confidence intervals for cause-specific mortality according to desired longevity (*n* = 39,902)

	Number of deaths	Desired longevity		

		Longer than	Average	Shorter than
Cardiovascular disease	1,505	1.00 (ref.)	0.98 (0.88–1.09)	1.11 (0.93–1.34)
Ischemic heart disease	516	1.00 (ref.)	0.99 (0.82–1.19)	1.13 (0.83–1.55)
Stroke	989	1.00 (ref.)	0.97 (0.85–1.11)	1.11 (0.88–1.39)
Cancer	3,281	1.00 (ref.)	1.01 (0.94–1.09)	1.14 (1.00–1.29)
Pneumonia	483	1.00 (ref.)	1.04 (0.86–1.26)	1.31 (0.94–1.82)
Accident	1,104	1.00 (ref.)	0.99 (0.87–1.13)	1.19 (0.98–1.46)
Suicide	178	1.00 (ref.)	1.23 (0.88–1.74)	2.15 (1.37–3.38)

Base model hazard ratio: adjusted for age (continuous variable), sex (men or women), marital status (married, divorced/widowed, or single), education (in school until age ≤15 years, age 16–18 years, or age ≥19 years).

The baseline characteristics according to the desired longevity categories did not change when we included those who had any history of cancer, stroke, or myocardial infarction (eTable 2 and eTable 3). There was also no change in the association between desired longevity and the risk of all-cause mortality or cause-specific mortality (eTable 4, eTable 5, and eTable 6). The results obtained after excluding all deaths that occurred within the first 2 years of follow-up showed an increase in the all-cause mortality risk in the “shorter than” group (eTable 7). eTable 8 shows the association between desired longevity and all-cause mortality after stratifying the participants according to past medical history. The results essentially remained unchanged from those in Table 2. Therefore, the association between desired longevity and mortality risk was not explained by medical history, health status, or reverse causality.

The association between desired longevity and all-cause mortality was significantly mediated by lifestyle behaviors (Table 5); smoking status by 17.4%, BMI by 4.4%, time spent walking by 4.1%, drinking status by 3.8%, and eating breakfast by 3.8%. Lifestyle behaviors overall significantly mediated this association by 30.4%.

**Table 5.** Mediation effects of lifestyle behaviors on the association between desired longevity and mortality (*n* = 39,902)

	Desired longevity			Proportion of Mediating Effect
	
	Longer than	Average	Shorter than	Shorter than
			
		HR (95% CI)	HR (95% CI)	(95% CI)
All-cause mortality				
Base model	1.00 (ref.)	1.00 (0.96–1.05)	1.12 (1.04–1.21)	
Base model + Body mass index^a^	1.00 (ref.)	1.01 (0.96–1.05)	1.12 (1.03–1.20)	4.4% (2.3–8.1%)^*^
Base model + Smoking status^b^	1.00 (ref.)	1.00 (0.96–1.05)	1.10 (1.02–1.18)	17.4% (10.7–27.1%)^*^
Base model + Drinking status^c^	1.00 (ref.)	1.00 (0.96–1.05)	1.12 (1.03–1.20)	3.8% (2.0–6.8%)^*^
Base model + Sleep duration^d^	1.00 (ref.)	1.01 (0.96–1.05)	1.12 (1.04–1.21)	1.2% (0.2–7.1%)
Base model + Time spent walking^e^	1.00 (ref.)	1.00 (0.96–1.05)	1.12 (1.03–1.20)	4.1% (2.4–7.0%)^*^
Base model + Eating breakfast^f^	1.00 (ref.)	1.01 (0.96–1.05)	1.12 (1.03–1.20)	3.8% (2.2–6.5%)^*^
Base model + All of lifestyle behaviors^g^	1.00 (ref.)	1.01 (0.96–1.05)	1.08 (1.00–1.17)	30.4% (19.1–44.6%)^*^

CI, confidence interval; HR, hazard ratio.

Base model HR: adjusted for age (continuous variable), sex (men or women), marital status (married, divorced/widowed, or single), education (in school until age ≤15 years, age 16–18 years, or age ≥19 years).

^a^Dummy variables for body mass index: <18.5, 18.5–24.9, ≥25.0 kg/m^2^ and missing.

^b^Dummy variables for smoking status: never smoking, ever smoking, and missing.

^c^Dummy variables for drinking status: never drinking, ever drinking, and missing.

^d^Dummy variables for sleep duration: ≤6 hours, 7–8 hours, ≥9 hours, and missing.

^e^Dummy variables for time spent walking: <1 h/day, ≥1 h/day, and missing.

^f^Dummy variables for eating breakfast: Yes, No, and missing.

^g^Combined ^a^ to ^f^.

^*^P < 0.001.

As a sensitivity analysis, we examined the mediating effects of lifestyle behaviors using the Sobel test (eFigure 1). Smoking status (*P* < 0.001), drinking status (*P* < 0.001), time spent walking (*P* = 0.016), and eating breakfast (*P* = 0.005) had significant mediating effects on the association between desired longevity and all-cause mortality. There was no change in the results after including participants who had a history of cancer, stroke, or myocardial infarction (eTable 9 and eFigure 2) or stratifying age and sex (data not shown).

We also performed mediation analyses for the association between desired longevity and mortality of cancer and suicide, respectively. The association between desired longevity and cancer mortality was significantly mediated by lifestyle behaviors (eTable 10). The proportion of mediation effects showed that smoking mediated the association by 15.7% and lifestyle behaviors overall mediated the association by 26.7%. According to the Sobel test, smoking status (*P* < 0.001), drinking status (*P* = 0.006), and eating breakfast (*P* = 0.030) had significant mediating effects on the association between desired longevity and cancer mortality (eFigure 3). In contrast, there was little mediating effect of lifestyle behaviors overall (2.8%) on the association between desired longevity and suicide mortality (eTable 10). No significant mediating effects of lifestyle behaviors was observed between desired longevity and suicide mortality based on the Sobel test (eFigure 4).

## DISCUSSION

In this population-based prospective cohort study that followed 39,902 middle-aged Japanese men and women from June 1990 to March 2015, we observed that shorter desired longevity was significantly associated with an increased risk of all-cause mortality, as well as mortalities from cancer and suicide. As much as 30.4% of the association between desired longevity and all-cause mortality was mediated by unhealthy lifestyles, such as smoking, obesity, and inactivity. To the best of our knowledge, this is the first study indicating that desired longevity of middle-aged participants (mean age: 51.7 years) well predicted survival over 25 years.

Only one prospective study has reported the association between desired longevity and mortality risk. In that study, longer desired longevity was associated with lower mortality risk, but the study focused only on older participants aged 75–90 years and the number of participants was small (*n* = 283).^17^ In addition, because desired longevity would be generally influenced by health status, the previous results may not be free from reverse causality.

We indicated that the association between desired longevity and mortality was consistently observed whether the participants were men or women, and aged in their 40s or 60s. In stratified analysis, the significant association might have disappeared as the number of participants decreased. The association between desired longevity and mortality would be independent of health status, because this association was observed even after excluding the deaths that occurred within the first 2 years of follow-up, including the participants who had a history of cancer, stroke, or myocardial infarction, and stratifying the participants by history of hypertension or diabetes.

The value of the mediation analysis might have been limited because the direct effect size was small in our study; however, the results appear reasonable. Mediation analyses indicated that lifestyle behaviors, especially smoking, could partly explain the association between desired longevity and all-cause mortality. Lifestyle behaviors particularly mediated the association between desired longevity and cancer mortality. The risk of cancer mortality was increased in participants with a shorter desired longevity because they were more prone to unhealthy lifestyle behaviors, but three quarters of the association remains unexplained. On the other hand, there were few mediating effects of lifestyle behaviors on the association between desired longevity and suicide mortality. It is suggested that shorter desired longevity could affect suicidal behavior because shorter desired longevity reflects little desire to live, which was indicated to be associated with increased risk of suicide in a previous study.^30^ It was surprising that this was an effect that lasted throughout the lifetime.

The results of the present study were similar to those of previous studies about positive psychologies. Positive psychologies, such as subjective sense of well-being,^11^^,^^12^ *ikigai* (life worth living),^13^ happiness,^14^ optimism,^15^ and enjoyment,^16^ are known to be associated with lower risk of morbidity and mortality by three pathways: 1) biological processes; 2) social factors; and 3) health behaviors.^31^ As biological processes, some explanatory mechanisms were suggested: positive psychologies (a) “may alter people’s susceptibility to disease by decreasing the activity of the sympathetic nervous system and increasing the activation of the parasympathetic nervous system”,^32^ and (b) “may reduce cortisol and stress-induced elevations of inflammatory and coagulation factors, such as fibrinogen and interleukin-6”.^33^^,^^34^ Positive psychologies can enhance social factors, such as social activity and relationships.^35^ Numerous studies have also found that positive psychologies were associated with healthy behaviors, such as smoking cessation, healthy dietary patterns, and regular exercise.^36^^,^^37^ On the other hand, shorter desired longevity could be associated with negative affectivity, which is known to increase the risk of mortality.^38^ Negative affectivity is governed by different neural pathways than positive psychologies,^39^ and is associated with poor mental and physical health status.^40^ Since our results showed that the mediating effect of lifestyle behaviors explains at most 30% of the association between desired longevity and mortality, other pathways of positive psychologies and negative affectivity may explain part of the rest.

The present study had some strengths. We dealt with a large number of residents in the community over a long time; the response rate to the baseline survey was 91.7%, and we followed 39,902 men and women for almost 25 years, with a follow-up rate of 91.0%. We controlled the effect of covariates and conducted stratified and sensitivity analyses so as to rule out reverse causality.

There were also some limitations to the present study. First, we did not collect information regarding self-related health, negative affectivity, such as depression, or any other questions about positive psychologies (eg, happiness) at baseline. Second, the validity and reliability of desired longevity score was not verified. Third, the results of the mediation analysis should be interpreted carefully because the direct effect was small. Fourth, desired longevity was assessed only once at baseline, and we have no data about how desired longevity changed over the follow-up. Fifth, we had no data on changes in the lifestyle behaviors of participants during the follow-up period. Lastly, because the participants were all Japanese, this study may have been affected by Japanese culture. Future studies in other populations and cultures are required.

According to an Internet survey of 1,500 Japanese in 2018, the average desired longevity was 77.1 years.^41^ This was shorter than the life expectancy in 2018, which was 81.1 years for men and 87.3 years for women. The survey also suggested that shorter desired longevity was associated with anxiety in old age, economic difficulty, illness and loss of autonomy, social isolation and solitude, and other factors. Achieving a society in which desired longevity approaches actual life expectancy is crucial to attaining well-being in aging. To realize a society where everyone wants to live a long life and where living a long life can be viewed in a positive light, comprehensive policies are needed to reduce physical, social, and financial anxiety in old age.

In conclusion, this population-based, prospective cohort study conducted in Japan has demonstrated that shorter desired longevity was significantly associated with an increased risk of all-cause mortality, as well as mortality from cancer and suicide. This association could be partly explained by unhealthy lifestyle behaviors, but other factors remain unknown. Desired longevity is an important topic for the society, and more detailed studies are needed that focus on the association between desired longevity and mortality.

## SUPPLEMENTARY MATERIAL

eTable 1. Baseline lifestyle characteristics according to the desired longevity stratified by sex (*n* = 39,902)

eTable 2. Demographic characteristics of sample according to the desired longevity after including those with history of cancer, stroke, and myocardial infarction (*n* = 41,758)

eTable 3. Baseline lifestyle characteristics according to the desired longevity stratified by sex after including those with history of cancer, stroke, and myocardial infarction (*n* = 41,758)

eTable 4. Hazard ratios and 95% confidence intervals for all-cause mortality according to desired longevity after including those with history of cancer, stroke, and myocardial infarction (*n* = 41,758)

eTable 5. Base model hazard ratios and 95% confidence intervals for all-cause mortality according to desired longevity stratified by age, sex, marital status, and education including those with history of cancer, stroke, and myocardial infarction (*n* = 41,758)

eTable 6. Base model hazard ratios and 95% confidence intervals for cause-specific mortality according to desired longevity including those with history of cancer, stroke, and myocardial infarction (*n* = 41,758)

eTable 7. Hazard ratios and 95% confidence intervals for all-cause mortality according to desired longevity after excluding all deaths within the first two years (*n* = 39,727)

eTable 8. Base model hazard ratios and 95% confidence intervals for all-cause mortality according to desired longevity stratified by past medical history

eTable 9. Mediation effects of lifestyle behaviors on the association between desired longevity and mortality including those with history of cancer, stroke, and myocardial infarction (*n* = 41,758)

eTable 10. Mediation effects of lifestyle behaviors on the association between desired longevity and mortality of cancer and suicide (*n* = 39,902)

eFigure 1. Lifestyle behavior models of the association between desired longevity and all-cause mortality using Sobel test

eFigure 2. Lifestyle behavior models of the association between desired longevity and all-cause mortality after including those with history of cancer, stroke, and myocardial infarction using Sobel test

eFigure 3. Lifestyle behavior models of the association between desired longevity and mortality of cancer using Sobel test

eFigure 4. Lifestyle behavior models of the association between desired longevity and mortality of suicide using Sobel test
