# Supplementary material for: How Long Would You Like to Live? A 25-year Prospective Observation of the Association Between Desired Longevity and Mortality
Source: J Epidemiol. 2023 Sep 5;33(9):464–70. doi: 10.2188/jea.JE20210493 (PMC10409531; doi:10.2188/jea.JE20210493)
Supplement: Supplementary file 1 [file je-33-464-s001.pdf]

eTable 1. Baseline lifestyle characteristics according to the desired longevity stratified by sex (*n*=39,902)

	Desired longevity			*P* value
	Longer than	Average	Shorter than	
**Men**				
Body mass index, %				0.19
<18.5 kg/m^2	2.1	1.8	2.7	
18.5–24.9 kg/m^2	70.6	70.4	70.1	
≥25.0 kg/m^2	27.3	27.8	27.2	
Ever smoker, %	80.6	81.1	84.2	0.004
Ever drinker, %	84.0	84.9	85.0	0.24
Sleep duration, %				
≤6 hours	15.9	17.1	22.2	<0.001
7–8 hours	73.4	74.6	68.3	
≥9 hours	10.7	8.3	9.5	
Time spent walking <1 h/day, %	52.6	56.6	56.4	<0.001
Not eating breakfast, %	6.9	7.5	13.8	<0.001
**Women**				
Body mass index, %				<0.001
<18.5 kg/m^2	2.9	2.4	3.1	
18.5–24.9 kg/m^2	64.1	66.4	68.2	
≥25.0 kg/m^2	33.0	31.2	28.7	
Ever smoker, %	10.3	10.3	14.7	<0.001
Ever drinker, %	27.1	29.9	35.8	<0.001
Sleep duration, %				<0.001
≤6 hours	20.9	22.8	27.9	
7–8 hours	71.3	71.5	67.3	
≥9 hours	7.8	5.7	4.8	
Time spent walking <1 h/day, %	50.8	55.0	57.7	<0.001
Not eating breakfast, %	6.1	5.8	8.0	<0.001

eTable 2. Demographic characteristics of sample according to the desired longevity after including those with history of cancer, stroke, and myocardial infarction (*n*=41,758)

	Desired longevity			***P*-value**
	**Longer than**	**Average**	**Shorter than**	
Number of participants	13,843	22,806	5,109	
Age, years, mean (SD)	52.8 (7.6)	51.7 (7.5)	50.0 (7.2)	<0.001
Men, %	56.3	47.4	34.1	<0.001
Marital status, %				<0.001
Married	89.9	90.9	86.9	
Divorced/widowed	6.7	6.7	8.7	
Single	3.4	2.4	4.4	
Education, %				<0.001
≤15 years	43.6	38.2	34.8	
16–18 years	44.3	47.3	50.0	
≥19 years	12.1	14.5	15.2	

SD, standard deviation.

eTable 3. Baseline lifestyle characteristics according to the desired longevity stratified by sex after including those with history of cancer, stroke, and myocardial infarction (*n*=41,758)

	Desired longevity			*P* value
	Longer than	Average	Shorter than	
**Men**				
Body mass index, %				0.061
<18.5 kg/m^2	2.2	1.9	2.9	
18.5–24.9 kg/m^2	70.5	70.5	70.1	
≥25.0 kg/m^2	27.3	27.6	27.0	
Ever smoker, %	80.9	81.4	84.3	0.005
Ever drinker, %	84.1	84.9	85.0	0.34
Sleep duration, %				
≤6 hours	15.8	16.8	22.2	<0.001
7–8 hours	73.1	74.5	68.1	
≥9 hours	11.1	8.7	9.8	
Time spent walking <1 h/day, %	53.0	56.7	56.6	<0.001
Not eating breakfast, %	6.7	7.3	13.6	<0.001
**Women**				
Body mass index, %				<0.001
<18.5 kg/m^2	3.0	2.5	3.3	
18.5–24.9 kg/m^2	63.9	66.3	68.1	
≥25.0 kg/m^2	33.1	31.2	28.6	
Ever smoker, %	10.2	10.3	14.8	<0.001
Ever drinker, %	27.0	29.8	36.0	<0.001
Sleep duration, %				<0.001
≤6 hours	20.9	22.7	28.1	
7–8 hours	71.2	71.4	66.9	
≥9 hours	8.0	6.0	5.0	
Time spent walking <1 h/day, %	51.1	55.1	58.1	<0.001
Not eating breakfast, %	6.0	5.7	8.0	<0.001

eTable 4. Hazard ratios and 95% confidence intervals for all-cause mortality according to desired longevity after including those with history of cancer, stroke, and myocardial infarction (*n*=41,758)

	Desired longevity		
	**Longer than**	**Average**	**Shorter than**
Number of participants	13,843	22,806	5,109
Person-year of follow-up	298,533	496,929	109,864
Number of deaths	3,737	5,178	967
Age and sex adjusted HR (95% CI)	1.00 (ref.)	0.99 (0.95–1.03)	1.11 (1.03–1.19)
Base model HR (95% CI)	1.00 (ref.)	0.99 (0.96–1.04)	1.11 (1.03–1.19)
Base model + history of cancer, stroke and myocardial infarction HR (95% CI)	1.00 (ref.)	0.99 (0.95–1.03)	1.09 (1.01–1.17)

CI, confidence interval; HR, hazard ratio.

Base model HR: adjusted for age (continuous variable), sex (men or women), marital status (married, divorced/widowed, or single), education (in school until age ≤15 years, age 16–18 years, or age ≥19 years).

eTable 5. Base model hazard ratios and 95% confidence intervals for all-cause mortality according to desired longevity stratified by age, sex, marital status, and education including those with history of cancer, stroke, and myocardial infarction (*n*=41,758)

	**Desired longevity**			***P* for interaction**
	**Longer than**	**Average**	**Shorter than**	
Age				
40–49 years	1.00 (ref.)	0.95 (0.85–1.05)	1.09 (0.94–1.27)	0.39
50–59 years	1.00 (ref.)	0.98 (0.92–1.05)	1.09 (0.97–1.21)	
≥60 years	1.00 (ref.)	1.04 (0.98–1.11)	1.12 (0.99–1.28)	
Sex				
Men	1.00 (ref.)	1.02 (0.97–1.08)	1.18 (1.07–1.31)	0.044
Women	1.00 (ref.)	0.95 (0.88–1.02)	1.02 (0.91–1.13)	
Marital status				
Married	1.00 (ref.)	0.98 (0.94–1.03)	1.09 (1.00–1.18)	0.22
Others[a]	1.00 (ref.)	1.07 (0.94–1.22)	1.26 (1.04–1.52)	
Education				
≤15 years	1.00 (ref.)	1.01 (0.95–1.08)	1.08 (0.97–1.21)	0.84
≥16 years	1.00 (ref.)	0.98 (0.93–1.05)	1.12 (1.01–1.23)	

[a]Others: divorced, widowed, or single

Base model hazard ratio: adjusted for the following variables (except for stratifying variables); age (continuous variable), sex (men or women), marital status (married, divorced/widowed, or single), education (in school until age ≤15 years, age 16–18 years, or age ≥19 years)

eTable 6. Base model hazard ratios and 95% confidence intervals for cause-specific mortality according to desired longevity including those with history of cancer, stroke, and myocardial infarction (*n*=41,758)

	Number of deaths	Desired longevity		
		**Longer than**	**Average**	**Shorter than**
Cardiovascular disease	1,702	1.00 (ref.)	0.96 (0.87–1.06)	1.12 (0.95–1.33)
Ischemic heart disease	576	1.00 (ref.)	0.93 (0.78–1.11)	1.11 (0.82–1.49)
Stroke	1,126	1.00 (ref.)	0.97 (0.86–1.10)	1.13 (0.92–1.40)
Cancer	3,627	1.00 (ref.)	1.01 (0.94–1.08)	1.12 (0.99–1.27)
Pneumonia	539	1.00 (ref.)	1.02 (0.86–1.23)	1.22 (0.89–1.68)
Accident	1,146	1.00 (ref.)	1.00 (0.88–1.14)	1.18 (0.97–1.44)
Suicide	183	1.00 (ref.)	1.22 (0.87–1.71)	2.05 (1.31–3.21)

Base model hazard ratio: adjusted for age (continuous variable), sex (men or women), marital status (married, divorced/widowed, or single), education (in school until age ≤15 years, age 16–18 years, or age ≥19 years).

eTable 7. Hazard ratios and 95% confidence intervals for all-cause mortality according to desired longevity after excluding all deaths within the first two years (*n*=39,727)

	**Desired longevity**		
	**Longer than**	**Average**	**Shorter than**
Number of participants	13,135	21,740	4,852
Person-year of follow-up	286,768	478,340	105,364
Number of deaths	3,333	4,628	862
Age and sex adjusted HR (95% CI)	1.00 (ref.)	0.99 (0.95–1.04)	1.12 (1.04–1.21)
Base model HR (95% CI)	1.00 (ref.)	1.00 (0.96–1.05)	1.11 (1.03–1.20)

CI, confidence interval; HR, hazard ratio.

Base model HR: adjusted for age (continuous variable), sex (men or women), marital status (married, divorced/widowed, or single), education (in school until age ≤15 years, age 16–18 years, or age ≥19 years).

eTable 8. Base model hazard ratios and 95% confidence intervals for all-cause mortality according to desired longevity stratified by past medical history

	Desired longevity		
	**Longer than**	**Average**	**Shorter than**
History of hypertension			
Yes	1.00 (ref.)	1.06 (0.97–1.16)	1.21 (1.05–1.40)
No	1.00 (ref.)	0.98 (0.93–1.03)	1.07 (0.98–1.17)
History of diabetes			
Yes	1.00 (ref.)	1.17 (0.99–1.37)	1.13 (0.86–1.48)
No	1.00 (ref.)	0.99 (0.94–1.04)	1.11 (1.03–1.20)
History of both hypertension and diabetes	1.00 (ref.)	1.17 (0.90–1.52)	1.01 (0.65–1.59)
History of neither hypertension nor diabetes	1.00 (ref.)	0.96 (0.91–1.02)	1.06 (0.97–1.16)

Base model hazard ratio: adjusted for age (continuous variable), sex (men or women), marital status (married, divorced/widowed, or single), education (in school until age ≤15 years, age 16–18 years, or age ≥19 years).

eTable 9. Mediation effects of lifestyle behaviors on the association between desired longevity and mortality including those with history of cancer, stroke, and myocardial infarction (*n*=41,758)

	Desired longevity			Proportion of Mediating Effect
	**Longer than**	**Average**	**Shorter than**	**Shorter than**
		HR (95% CI)	HR (95% CI)	(95% CI)
All-cause mortality				
Base model	1.00 (ref.)	0.99 (0.96–1.04)	1.11 (1.03–1.19)	
Base model + Body mass index[a]	1.00 (ref.)	0.99 (0.96–1.04)	1.10 (1.02–1.18)	5.4% (2.8–10.3%)*
Base model + Smoking status[b]	1.00 (ref.)	0.99 (0.95–1.04)	1.08 (1.01–1.17)	19.6% (11.7–30.9%)*
Base model + Drinking status[c]	1.00 (ref.)	0.99 (0.95–1.04)	1.10 (1.02–1.18)	4.4% (2.4–7.9%)*
Base model + Sleep duration[d]	1.00 (ref.)	1.00 (0.96–1.04)	1.11 (1.03–1.19)	1.1% (1.1–1.2%)
Base model + Time spent walking[e]	1.00 (ref.)	0.99 (0.95–1.04)	1.10 (1.02–1.18)	5.9% (3.4–10.0%)*
Base model + Eating breakfast[f]	1.00 (ref.)	0.99 (0.96–1.04)	1.10 (1.02–1.18)	4.1% (2.4–7.2%)*
Base model + All of lifestyle behaviors[g]	1.00 (ref.)	0.99 (0.96–1.04)	1.07 (0.99–1.15)	35.5% (21.4–52.5%)*

CI, confidence interval; HR, hazard ratio.

Base model HR: adjusted for age (continuous variable), sex (men or women), marital status (married, divorced/widowed, or single), education (in school until age ≤15 years, age 16–18 years, or age ≥19 years).

[a]Dummy variables for body mass index: <18.5, 18.5–24.9, ≥25.0 kg/m^2 and missing.

[b]Dummy variables for smoking status: never smoking, ever smoking, and missing.

[c]Dummy variables for drinking status: never drinking, ever drinking, and missing.

[d]Dummy variables for sleep duration: ≤6 hours, 7–8 hours, ≥9 hours, and missing.

[e]Dummy variables for time spent walking: <1 h/day, ≥1 h/day, and missing.

[f]Dummy variables for eating breakfast: Yes, No, and missing.

[g]Combined [a] to [f].

*P<0.001.

eTable 10. Mediation effects of lifestyle behaviors on the association between desired longevity and mortality of cancer and suicide (*n*=39,902)

	Desired longevity			Proportion of Mediating Effect
	**Longer than**	**Average**	**Shorter than**	**Shorter than**
		HR (95% CI)	HR (95% CI)	(95% CI)
**Cancer**				
Base model	1.00 (ref.)	1.01 (0.94–1.09)	1.14 (1.00–1.29)	
Base model + Body mass index[a]	1.00 (ref.)	1.01 (0.94–1.09)	1.13 (0.99–1.28)	2.4% (1.0–5.8%)*
Base model + Smoking status[b]	1.00 (ref.)	1.01 (0.94–1.09)	1.11 (0.98–1.26)	15.7% (8.0–28.4%)**
Base model + Drinking status[c]	1.00 (ref.)	1.01 (0.94–1.09)	1.13 (0.99–1.28)	3.5% (1.6–7.4%)**
Base model + Sleep duration[d]	1.00 (ref.)	1.01 (0.94–1.09)	1.13 (0.99–1.28)	2.1% (0.8–5.2%)*
Base model + Time spent walking[e]	1.00 (ref.)	1.01 (0.94–1.09)	1.13 (0.99–1.28)	2.7% (1.1–6.5%)*
Base model + Eating breakfast[f]	1.00 (ref.)	1.02 (0.94–1.09)	1.13 (0.99–1.28)	3.9% (1.9–8.0%)**
Base model + All of lifestyle behaviors[g]	1.00 (ref.)	1.01 (0.94–1.09)	1.10 (0.97–1.25)	26.7% (13.6–45.7%)**
**Suicide**				
Base model	1.00 (ref.)	1.23 (0.88–1.74)	2.15 (1.37–3.38)	
Base model + Body mass index[a]	1.00 (ref.)	1.24 (0.88–1.74)	2.15 (1.37–3.37)	n/a
Base model + Smoking status[b]	1.00 (re.)	1.23 (0.87–1.73)	2.12 (1.35–3.33)	2.0% (1.1–3.3%)**
Base model + Drinking status[c]	1.00 (ref.)	1.23 (0.88–1.74)	2.15 (1.37–3.38)	n/a
Base model + Sleep duration[d]	1.00 (ref.)	1.23 (0.88–1.74)	2.14 (1.36–3.36)	n/a
Base model + Time spent walking[e]	1.00 (ref.)	1.23 (0.88–1.73)	2.16 (1.37–3.39)	n/a
Base model + Eating breakfast[f]	1.00 (ref.)	1.23 (0.87–1.73)	2.16 (1.37–3.38)	n/a
Base model + All of lifestyle behaviors[g]	1.00 (ref.)	1.23 (0.87–1.73)	2.11 (1.34–3.31)	2.8% (1.3–6.2%)*

CI, confidence interval; HR, hazard ratio; n/a, not applicable.

Base model HR: adjusted for age (continuous variable), sex (men or women), marital status (married, divorced/widowed, or single), education (in school until age ≤15 years, age 16–18 years, or age ≥19 years).

[a]Dummy variables for body mass index: <18.5, 18.5–24.9, ≥25.0 kg/m^2 and missing.

[b]Dummy variables for smoking status: never smoking, ever smoking, and missing.

[c]Dummy variables for drinking status: never drinking, ever drinking, and missing.

[d]Dummy variables for sleep duration: ≤6 hours, 7–8 hours, ≥9 hours, and missing.

[e]Dummy variables for time spent walking: <1 h/day, ≥1 h/day, and missing.

[f]Dummy variables for eating breakfast: Yes, No, and missing.

[g]Combined [a] to [f].

[*]*P*<0.005.

[**]*P*<0.001.

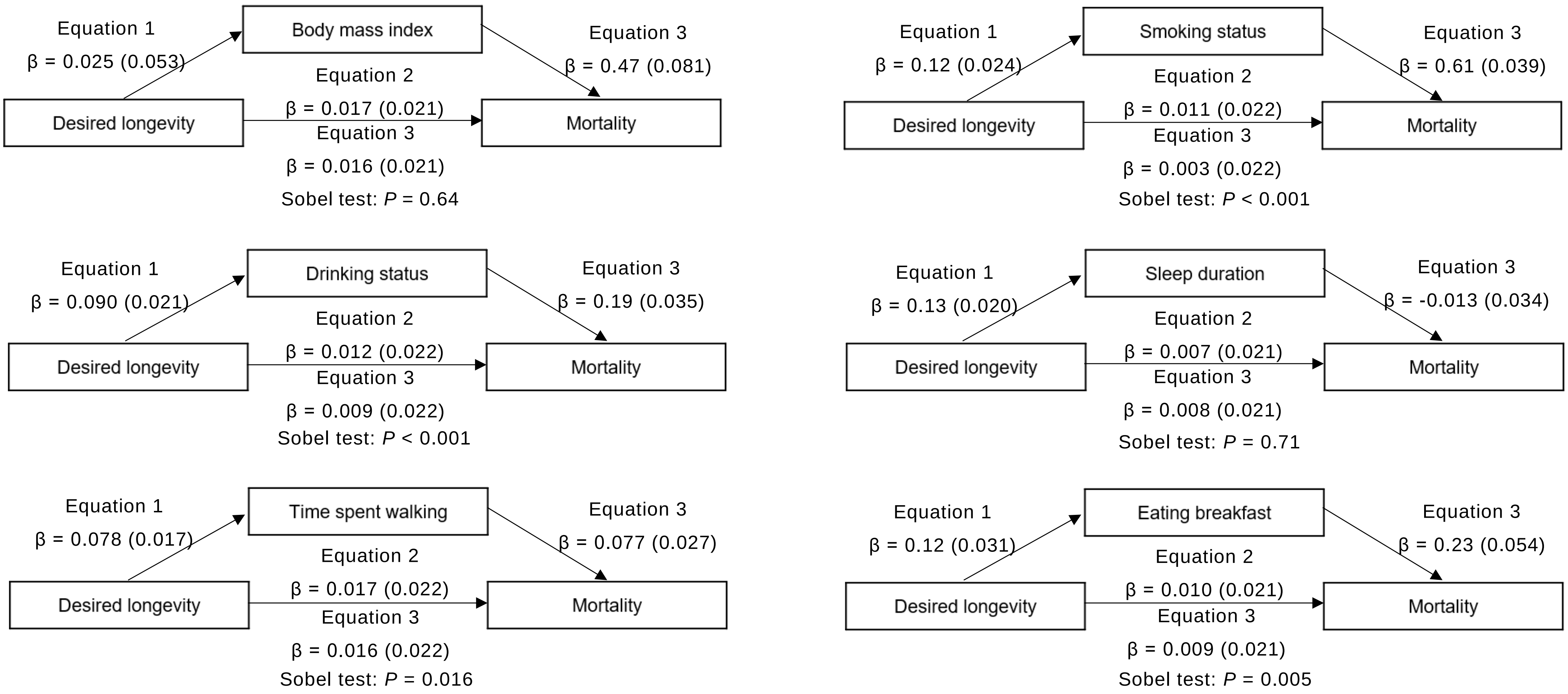

eFigure 1. Lifestyle behavior models of the association between desired longevity and all-cause mortality using Sobel test

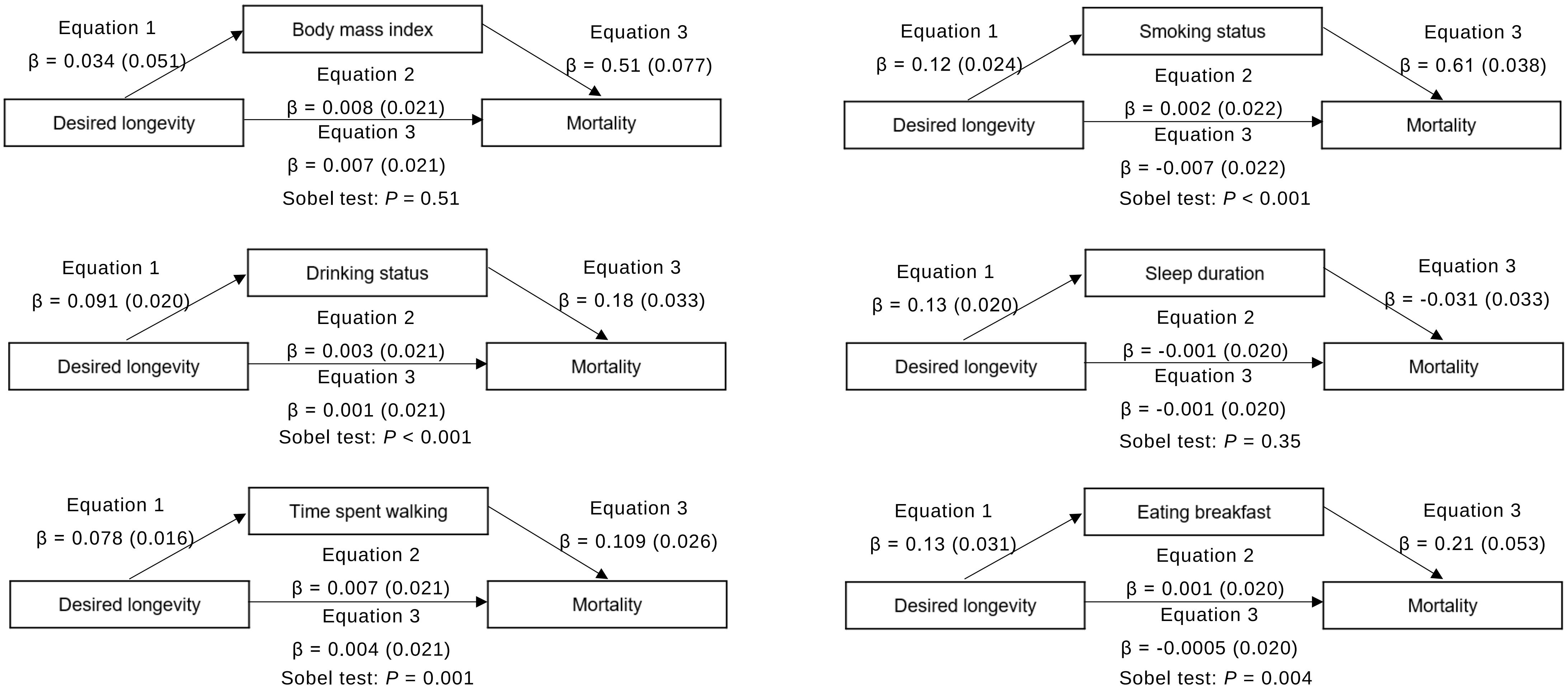

eFigure 2. Lifestyle behavior models of the association between desired longevity and all-cause mortality after including those with history of cancer, stroke, and myocardial infarction using Sobel test

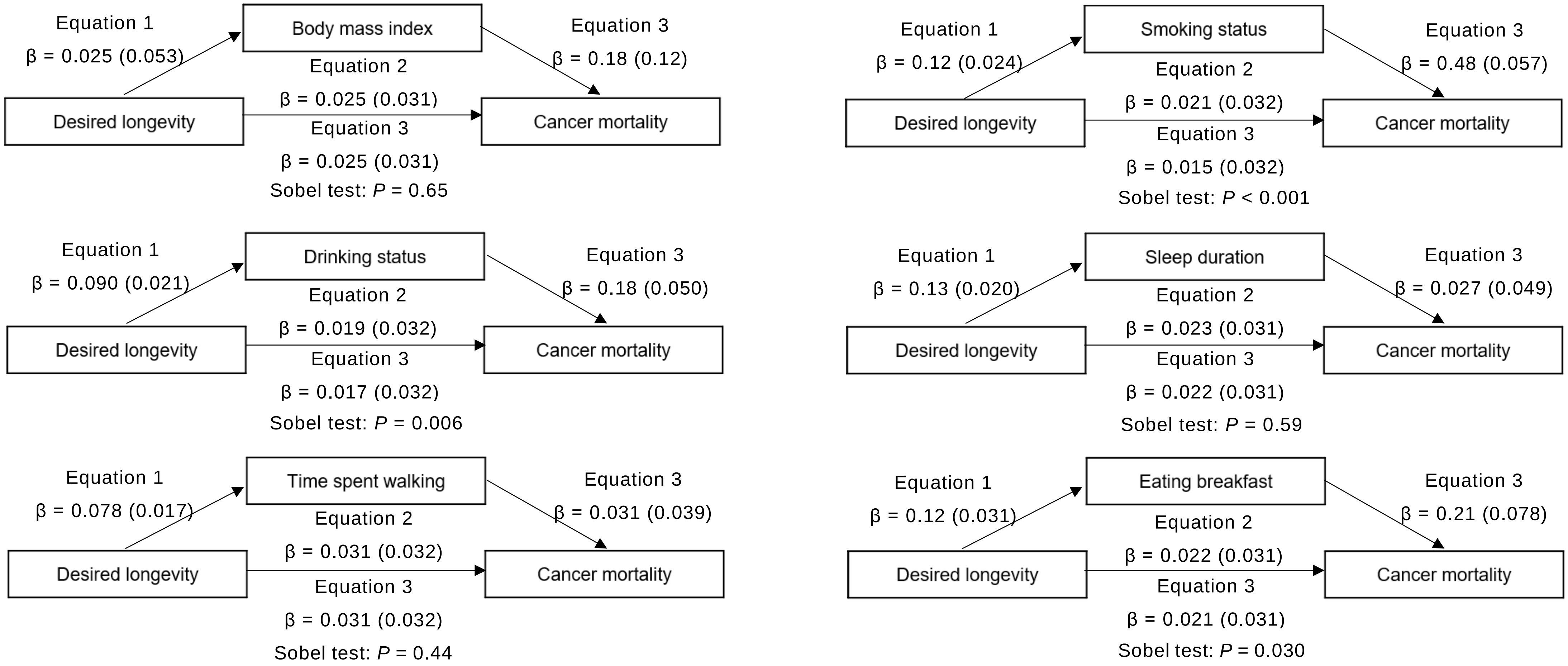

eFigure 3. Lifestyle behavior models of the association between desired longevity and mortality of cancer using Sobel test

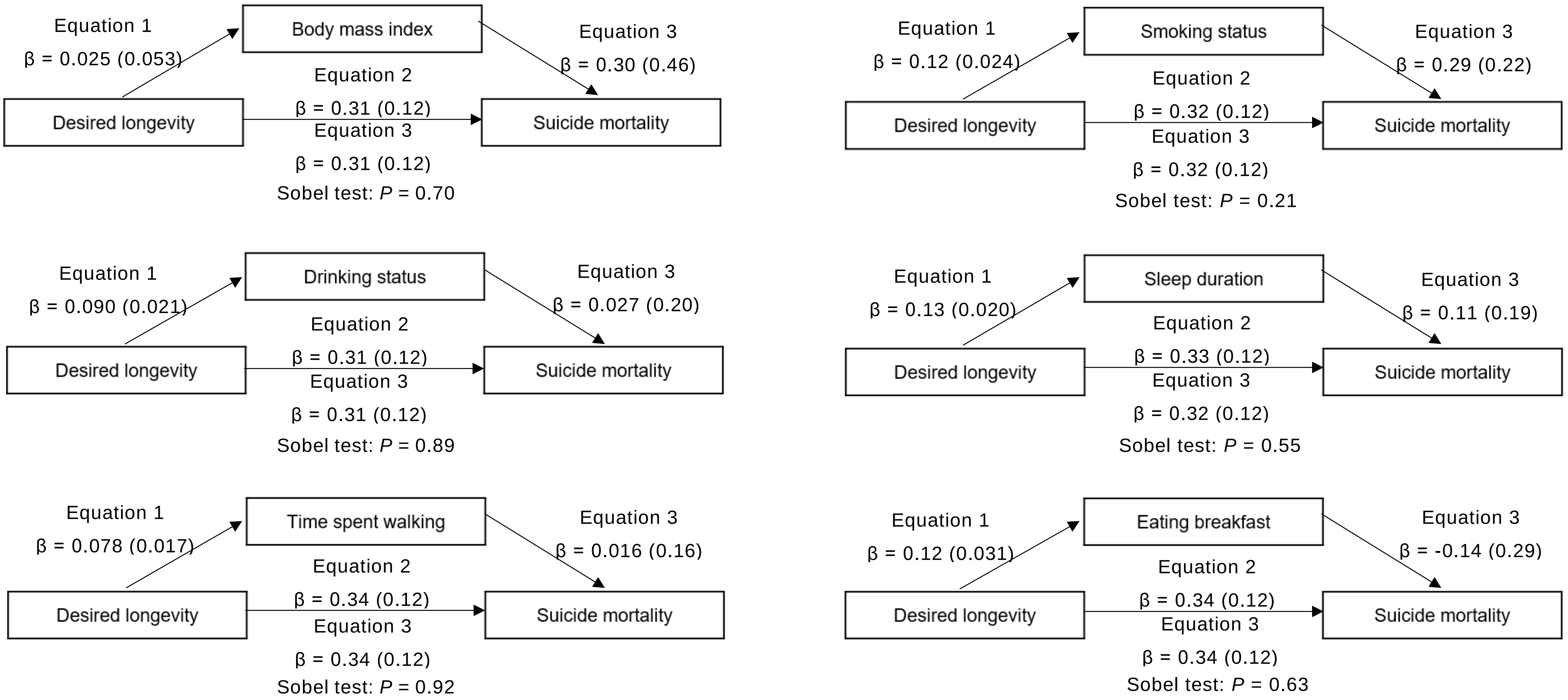

eFigure 4. Lifestyle behavior models of the association between desired longevity and mortality of suicide using Sobel test
